# Risk Factors for Death or Cardiovascular Events after Acute Coronary Syndrome in Patients with Myeloproliferative Neoplasms

**DOI:** 10.3390/hematolrep15020040

**Published:** 2023-06-07

**Authors:** Orly Leiva, Andrew Jenkins, Rachel P. Rosovsky, Rebecca K. Leaf, Katayoon Goodarzi, Gabriela Hobbs

**Affiliations:** 1 Division of Cardiovascular Medicine, Department of Medicine, New York University Langone Health, New York, NY 10016, USA; 2 Department of Medicine, Brigham and Women’s Hospital and Harvard Medical School, Boston, MA 02115, USA; 3 Division of Hematology and Oncology, Department of Medicine, Massachusetts General Hospital and Harvard Medical School, Boston, MA 02115, USA

**Keywords:** myeloproliferative neoplasms, thrombosis, myocardial infarction, outcomes

## Abstract

Patients with myeloproliferative neoplasms (MPNs) are at increased risk of cardiovascular disease (CVD), including acute coronary syndrome (ACS). However, data on long-term outcomes of patients with MPN who have had ACS and risk factors for all-cause death or CV events post-ACS hospitalization are lacking. We conducted a single-center study of 41 consecutive patients with MPN with ACS hospitalization after MPN diagnosis. After a median follow-up of 80 months after ACS hospitalization, 31 (76%) experienced death or a CV event (myocardial infarction, ischemic stroke, or heart failure hospitalization). After multivariable Cox proportional hazards regression, index ACS within 12 months of MPN diagnosis (HR 3.84, 95% CI 1.44–10.19), WBC ≥ 20 K/µL (HR 9.10, 95% CI 2.71–30.52), *JAK2* mutation (HR 3.71, 95% CI 1.22–11.22), and prior CVD (HR 2.60, 95% CI 1.12–6.08) were associated with increased death or CV events. Further studies are warranted to improve cardiovascular outcomes in this patient population.

## 1. Introduction

Myeloproliferative neoplasms (MPNs), including polycythemia vera (PV), essential thrombocytosis (ET), and myelofibrosis (MF), are clonal stem cell neoplasms associated with increased risk of cardiovascular disease (CVD), including acute coronary syndrome (ACS), heart failure (HF), thrombosis, and bleeding [1]. Driver mutations in the pro-inflammatory JAK-STAT signaling pathway are present in most MPN patients, and *JAK2* mutations are the most prevalent. Clonal hematopoiesis of indeterminate potential (CHIP) is a disorder of clonal hematopoiesis without alterations in blood counts and is also associated with increased cardiovascular events. Furthermore, among patients with CHIP, carriers of *JAK2* mutations are at the highest risk of cardiovascular events [2]. Experimental studies have shown accelerated atherosclerosis in *Jak2V617F*-mutated mouse models [3]. Additionally, MPN patients with *JAK2* mutations have an increased risk of arterial thrombosis compared with patients without *JAK2* mutations [4,5]. There is a paucity of data on outcomes post-ACS hospitalization in patients with MPN. Additionally, given the increased risk of death or cardiovascular (CV) events (thrombosis and HF) in patients with MPN, investigations into risk factor identification after ACS in this high-risk patient population are warranted. Therefore, this study aimed to describe the incidence of death or CV events after ACS in patients with MPN.

## 2. Materials and Methods

### 2.1. Patient Population

We conducted a single-center, retrospective observational cohort study using data gathered through the electronic medical record (EMR) within Massachusetts General Hospital. We identified 41 consecutive patients aged 18 years or older who met World Health Organization diagnostic criteria for MPNs (ET, PV, and MF) who were admitted to the hospital for ACS after being diagnosed with an MPN between 1 January 2000 and 1 January 2020 [6]. If patients progressed to secondary MF prior to ACS hospitalization, they were classified as MF. Acute coronary syndrome includes non-ST elevation ACS (NSTE-ACS) and ST elevation ACS (STE-ACS) per the fourth universal definition of myocardial infarction [7]. Treatment of ACS during index event was captured, including percutaneous coronary intervention (PCI), coronary artery bypass grafting (CABG), or medical therapy. Patient demographics, baseline characteristics, co-morbidities at time of index ACS event, medications (aspirin, P2Y12 inhibitor, statin, and anticoagulation) at discharge or within 12 months after index ACS event, and complete blood count at index ACS event were gathered. This study was approved, and requirement of informed consent were waived by the Institutional Review Board of Massachusetts General Hospital.

### 2.2. Outcomes and Statistical Analysis

Our primary outcome was death or CV event. CV event was a composite of myocardial infarction (MI), ischemic stroke, and heart failure (HF) hospitalization. Categorical variables were presented by count and percentages; continuous variables were presented as median and interquartile ranges (IQR). Patients with MPN admitted for ACS who experienced death or CV events were compared with patients without death or CV events to identify MPN-specific risk factors for death or CV events. MPN-specific variables that were statistically significant (*p* value < 0.05) between patients with and without death or CV events were used as co-variables in a multivariable Cox proportional hazards regression model. Multivariable Cox proportional hazards regression modeling was also adjusted for prior cardiovascular disease (AF, HF, coronary artery disease, and stroke). Continuous variables were compared using Mann–Whitney test. Categorical variables were assessed using Fisher’s Exact Test. A two-sided *p* value of < 0.05 was considered significant. Statistical analysis was performed using Stata version 15.1 (StataCorp LLC, College Station, TX, USA).

## 3. Results

A total of 41 patients (15 with PV, 19 with ET, and 7 with MF) were included, of whom 39 had NSTE-ACS and 2 had STE-ACS. Revascularization was performed in 23 (56%) of patients. Percutaneous coronary intervention (PCI) was performed in 16 (39%), and coronary artery bypass-grafting (CABG) in 7 (17%) of the patients. The median age at the time of index ACS was 74 years (IQR 67, 84), and the median time from MPN diagnosis to the index ACS event was 47 months (IQR 12, 84). Thirty-one (76%) patients experienced death or a CV event with a median follow-up time of 80 months (IQR 27, 98). The prevalence of death or CV events at 12 and 24 months was 32% and 37%, respectively. There was no difference in age at index ACS event (median 74 vs. 74 years, *p* = 0.27), follow-up time (median 80 vs. 67 months, *p* = 0.67), body mass index (median 23 vs. 23, *p* = 0.22), sex (68% vs. 50% male, *p* = 0.26), or co-morbidities between patients with death or CV events and those without. Patients experiencing death or a CV event had a shorter median time from MPN diagnosis to the index ACS event compared with patients without (32 month vs. 82, *p* = 0.029). More patients experiencing death or a CV event were positive for *JAK2V617F* (84% vs. 40%, *p* = 0.013). Patients experiencing death or a CV event had higher white blood cell counts (WBC) at the index event compared with patients without (14 K/µL vs. 8 K/µL, *p* = 0.008). There were no differences in the hematocrit (41% vs. 38%, *p* = 0.53) or platelet count (429 K/µL vs. 420 K/µL, *p* = 0.86). There was no difference in MPN types or MPN treatment between patients who did and did not experience death or a CV event. There were also no differences in any revascularization rates (61% vs. 40%, *p* = 0.29) at the index ACS event between patients who did and did not experience death or a CV event. Patient characteristics are summarized in Table 1.

**Figure 1:**
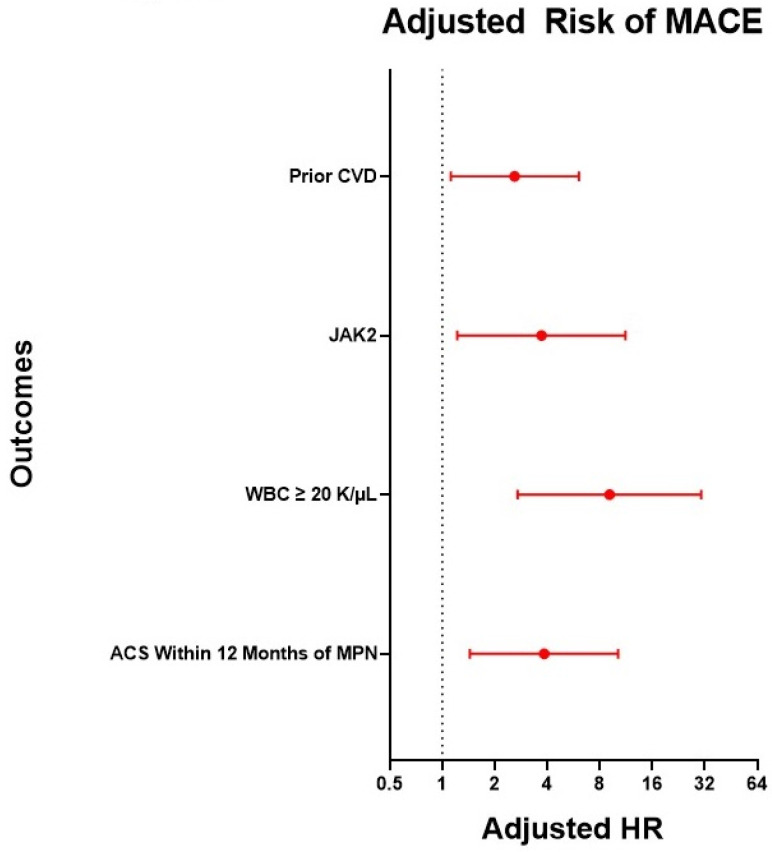
Risk factors associated with increased risk of death or CV event. Forrest plot of adjusted hazards ratio (HR) for death or CV event after multivariable Cox proportional hazards regression. Index ACS within 12 months of MPN diagnosis (HR 3.84, 95% CI 1.44–10.19), WBC ≥ 20 K/µL (HR 9.10, 95% CI 2.71–30.52), *JAK2* mutation (HR 3.71, 95% CI 1.22–11.22), and prior CVD (HR 2.60, 95% CI 1.12–6.08) were associated with increased death or CV event.

To assess the risk of death or a CV event, we performed a multivariable Cox proportional hazard using time from the MPN diagnosis to the index ACS event, WBC, *JAK2* status, and prior CVD as the co-variables. For routine clinical use, a WBC continuous variable was categorized as WBC < 10 K/µL, WBC 10 K/µL to <20 K/µL, and WBC ≥ 20 K/µL. Additionally, given that the risk of arterial thrombosis in MPNs is highest within 1 year of diagnosis of MPN, we categorized the time from MPN diagnosis to the index ACS event variable to ≤ 12 months or > 12 months [8]. After multivariable Cox proportional hazards regression, index ACS within 12 months of MPN diagnosis (HR 3.84, 95% CI 1.44–10.19), WBC ≥ 20 K/µL (HR 9.10, 95% CI 2.71–30.52), *JAK2* mutation (HR 3.71, 95% CI 1.22–11.22), and prior CVD (HR 2.60, 95% CI 1.12–6.08) were associated with an increased risk of death or CV event, see Figure 1.

## 4. Discussion

Arterial thrombosis, including ACS, is a complication of MPNs and is associated with significant morbidity and mortality. In our cohort, patients with MPN had a significant burden of MACE, with 76% of patients with MPN and ACS experiencing death or a CV event at any time and 32% having MACE within 12 months of ACS. While there is a paucity of studies comparing MPN versus non-MPN patient populations post-ACS, the 32% one-year death or CV rate is higher than 12-month post-ACS outcomes in the general population, which range from 7.5% to 12.1%, depending on the study and definition of the CV event [9,10,11]. Additionally, our study identified the potential risk factors for death or CV events in patients with MPN, including leukocytosis, *JAK2* mutation, ACS events within 12 months of MPN diagnosis, and prior CVD. In the general population, leukocytosis has also been associated with an increased risk of cardiovascular death and heart failure after ACS, particularly in STE-ACS, and is generally thought to be due to an inflammatory response to myocardial necrosis [12,13,14]. In our cohort, the median WBC was higher than what is described in the literature in the general ACS population (11 in our cohort vs. 7 to 10 K/µL) [12,13,14]. Additionally, most of the patients in our study had NSTE-ACS. Therefore, leukocytosis may represent an exaggerated inflammatory response to myocardial necrosis or uncontrolled MPN disease at the time of index hospitalization. In agreement with prior studies, *JAK2* mutation, prior CVD, and early post-MPN diagnosis have been associated with increased risks of arterial thrombosis in MPNs in prior studies [4,5,8,15]. Our results suggest that patients with MPN who present with ACS and have a history of *JAK2* mutation, prior CVD, have recently been diagnosed with MPN, and have leukocytosis at the time of ACS should be monitored closely for adverse cardiac events.

Our study did not show a difference in death or CV events between patients who were on DAPT for one year, though our sample size was too small to draw any conclusions. The length of DAPT therapy in this patient population is an important unanswered question, as the risk of bleeding and recurrent CV events must be weighed. The current guidelines recommend DAPT for at least 12 months after PCI for ACS [16]. However, the duration of DAPT has not been thoroughly evaluated in patients with MPN. Additionally, patients with MPN are at risk of venous thromboembolism or atrial fibrillation, which may necessitate treatment with anticoagulation. Indeed, in one study of patients with PV, treatment with both aspirin and anticoagulation was associated with a significantly increased risk of bleeding [17]. This highlights the need for further studies to investigate the optimal duration of DAPT and antithrombotic strategies in patients with MPN and ACS.

Limitations of our study include its small sample size, single-center, and retrospective nature. Additionally, while the P2Y12 inhibitor and anticoagulation use were captured, the type and dose of the P2Y12 inhibitor and anticoagulant used were not. Patients with MPN are at risk of both cardiovascular and non-cardiovascular etiologies of death, including disease progression. Leukocytosis and *JAK2* mutation are two risk factors in our study that are also associated with disease progression in MPNs; thus, it is possible that these risk factors contribute to death or CV events through non-cardiovascular etiologies [18,19]. Additionally, we included all-cause death as part of our outcome of interest, and, therefore, the contribution of *JAK2* mutation and WBC on CV-specific death is unclear, given that these variables are also risk factors for non-CV death, including transformation to acute leukemia or myelofibrosis. Our study had too few patients and events to evaluate the role of MPN-specific therapy, including cytoreductive strategies, on outcomes post-ACS. Future studies with larger sample sizes are needed in order to investigate the role of cytoreduction post-ACS in this patient population. However, our findings are hypothesis-generating, which will hopefully spur further investigation in order to improve outcomes in this high-risk population.

## 5. Conclusions

Patients with MPN who had a hospitalization for ACS are at increased risk of MACE post-hospitalization. Index ACS events within 12 months of MPN diagnosis, leukocytosis at the time of index ACS events, prior CVD, and *JAK2* mutations were associated with an increased risk of MACE. Patients with recently diagnosed MPN should be monitored carefully for atherosclerotic cardiovascular disease, and risk factors should be optimized in this patient population. Further studies, including larger multi-centered registry studies, are needed in order to further elucidate the risk factors for adverse cardiac events in this patient population.

## Figures and Tables

**Table 1:** Characteristics of patients who did and did not experience death or cardiovascular event.

	AllN = 41	Death or CV EventN = 31	No Death or CV EventN = 10	*p* Value
Patient characteristics				
Age at index, median (IQR)	74 (67, 84)	74 (64, 83)	74 (68, 90)	0.27
Median time from MPN to index, months (IQR)	47 (12, 84)	32 (8, 75)	82 (55, 107)	0.029
MedianfFollow-up, months (IQR)	80 (27, 98)	80 (27, 100)	67 (13, 93)	0.68
BMI (IQR)	23 (19, 26)	23 (19, 27)	23 (20, 26)	0.22
Male, N (%)	26 (63)	21 (68)	5 (50)	0.26
MPN characteristics				
Age at MPN, median (IQR)	68 (63, 79)	71 (63, 79)	68 (63, 81)	0.95
Type of MPN, N (%)				
PV	15 (37)	12 (39)	3 (30)	0.70
ET	19 (46)	13 (42)	6 (60)	
MF	7 (17)	6 (19)	1 (10)	
*JAK2V617F* Positive, N (%)	30 (73)	26 (84)	4 (40)	0.013
*MPL* Positive, N (%)	1 (2.4)	1 (3.2)	0	1.00
*CALR* Positive, N (%)	3 (7.3)	2 (6.4)	1 (10.0)	1.00
Treatment for MPN, N (%)	38 (93)	29 (94)	9 (90)	1.00
Hydroxyurea	30 (73)	22 (71)	8 (80)	0.70
Ruxolitinib	7 (17)	3 (16)	2 (20)	1.00
Phlebotomy	13 (31.7)	2 (20.0)	11 (35.5)	0.46
Anagrelide	4 (9.8)	2 (20.0)	2 (6.4)	0.24
Co-Morbidities, N (%)				
Prior CVD ^a^	24 (59)	17 (55)	7 (70)	0.48
CAD	12 (29)	9 (29)	3 (30)	1.00
Prior bleed	10 (24)	7 (29)	3 (18)	0.48
DM	5 (12)	4 (13)	1 (10)	1.00
HTN	31 (76)	24 (77)	7 (70)	0.68
Prior stroke/TIA	8 (20)	8 (26)	0	0.16
Atrial fibrillation	12 (29)	8 (26)	4 (40)	0.44
HF	15 (37)	13 (42)	2 (20)	0.28
Never smoking	13 (32)	11 (35)	2 (20)	0.46
Medications 1 year post-index, N (%)				
Aspirin	37 (90)	28 (90)	9 (90)	1.00
P2Y12 inhibitor	19 (46)	15 (48)	4 (40)	0.73
DAPT	19 (46)	15 (48)	4 (40)	0.73
Anticoagulation	12 (29)	10 (32)	2 (20)	0.69
Statin	30 (73)	25 (81)	5 (50)	0.098
Labs at index				
WBC (K/µL), median (IQR)	11 (8, 20)	14 (9, 25)	8 (8, 11)	0.008
Hematocrit (%), median (IQR)	39 (32, 42)	41 (32, 43)	38 (32, 41)	0.53
Platelets (K/µL), median (IQR)	429 (311, 608)	429 (266, 708)	420 (347, 607)	0.86
Spleen size (cm), median (IQR)	13 (11, 15)	14 (12, 16)	11 (8, 14)	0.065
Type of event				
STE-ACS	2 (5)	2 (6)	0	1.00
NSTE-ACS	39 (95)	29 (94)	10 (100)	1.00
LVEF at index, median (IQR)	58 (43, 68)	55 (47, 70)	60 (45, 68)	0.98
Treatment, N (%)				
PCI	16 (39)	13 (42)	3 (30)	0.71
DES	8	7	1	N/A
BMS	6	4	2	N/A
POBA	1	1	0	N/A
Unknown	1	1	0	N/A
CABG	7 (17)	6 (19)	1 (10)	0.66
Any revascularization	23 (56)	19 (61)	4 (40)	0.29
Outcomes				
Acute coronary syndrome	11 (27)	11 (35)	0	N/A
Ischemic stroke	6 (15)	6 (19)	0	N/A
HF hospitalization	17 (41)	17 (55)	0	N/A
All-cause death	17 (41)	17 (55)	0	N/A
CV-related death	8 (20)	8 (26)	0	N/A

Abbreviations: ACS, acute coronary syndrome; BMS, bare metal stent; CABG, coronary artery bypass grafting; CAD, coronary artery disease; CV, cardiovascular; CVD, cardiovascular disease; DAPT, dual antiplatelet therapy; DES, drug-eluting stent; DM, diabetes mellitus; ET, essential thrombocytosis; HF, heart failure; HTN, hypertension; IQR, interquartile range; LVEF, left ventricular ejection fraction; MF, myelofibrosis; MPN, myeloproliferative neoplasm; MRA, mineralocorticoid antagonist; NSTE-ACS, non-ST-elevation acute coronary syndrome; PCI, percutaneous coronary intervention; PH, pulmonary hypertension; POBA, plain old balloon angioplasty; PV, polycythemia vera; STE-ACS, ST-elevation acute coronary syndrome; TIA, transient ischemic attack; WBC, white blood cell count. ^a^ Prior CVD includes prior CAD, peripheral vascular disease, heart failure, stroke or atrial fibrillation.

## Data Availability

Data is available upon reasonable request to the corresponding author.

## References

[1] Leiva O., Hobbs G., Ravid K., Libby P. (2022). Cardiovascular Disease in Myeloproliferative Neoplasms. JACC CardioOncol..

[2] Jaiswal S., Natarajan P., Silver A.J., Gibson C.J., Bick A.G., Shvartz E., McConkey M., Gupta N., Gabriel S., Ardissino D. (2017). Clonal Hematopoiesis and Risk of Atherosclerotic Cardiovascular Disease. N. Engl. J. Med..

[3] Sano S., Wang Y., Yura Y., Sano M., Oshima K., Yang Y., Katanasaka Y., Min K.D., Matsuura S., Ravid K. (2019). JAK2 (V617F) -Mediated Clonal Hematopoiesis Accelerates Pathological Remodeling in Murine Heart Failure. JACC Basic Transl. Sci..

[4] Carobbio A., Thiele J., Passamonti F., Rumi E., Ruggeri M., Rodeghiero F., Randi M.L., Bertozzi I., Vannucchi A.M., Antonioli E. (2011). Risk factors for arterial and venous thrombosis in WHO-defined essential thrombocythemia: An international study of 891 patients. Blood.

[5] Cerquozzi S., Barraco D., Lasho T., Finke C., Hanson C.A., Ketterling R.P., Pardanani A., Gangat N., Tefferi A. (2017). Risk factors for arterial versus venous thrombosis in polycythemia vera: A single center experience in 587 patients. Blood Cancer J..

[6] Arber D.A., Orazi A., Hasserjian R.P., Borowitz M.J., Calvo K.R., Kvasnicka H.M., Wang S.A., Bagg A., Barbui T., Branford S. (2022). International Consensus Classification of Myeloid Neoplasms and Acute Leukemias: Integrating morphologic, clinical, and genomic data. Blood.

[7] Thygesen K., Alpert J.S., Jaffe A.S., Chaitman B.R., Bax J.J., Morrow D.A., White H.D., Executive Group on behalf of the Joint European Society of Cardiology, American College of Cardiology, American Heart Association (2018). Fourth Universal Definition of Myocardial Infarction (2018). Circulation.

[8] Hultcrantz M., Bjorkholm M., Dickman P.W., Landgren O., Derolf A.R., Kristinsson S.Y., Andersson T.M.L. (2018). Risk for Arterial and Venous Thrombosis in Patients with Myeloproliferative Neoplasms: A Population-Based Cohort Study. Ann. Intern. Med..

[9] Wiviott S.D., Braunwald E., McCabe C.H., Montalescot G., Ruzyllo W., Gottlieb S., Neumann F.J., Ardissino D., De Servi S., Murphy S.A. (2007). Prasugrel versus clopidogrel in patients with acute coronary syndromes. N. Engl. J. Med..

[10] Montalescot G., Dallongeville J., Van Belle E., Rouanet S., Baulac C., Degrandsart A., Vicaut E. (2007). STEMI and NSTEMI: Are they so different? 1 year outcomes in acute myocardial infarction as defined by the ESC/ACC definition (the OPERA registry). Eur. Heart J..

[11] Pendyala L.K., Torguson R., Loh J.P., Kitabata H., Minha S., Badr S., Dvir D., Barbash I.M., Satler L.F., Pichard A.D. (2013). Comparison of adverse outcomes after contemporary percutaneous coronary intervention in women versus men with acute coronary syndrome. Am. J. Cardiol..

[12] O’Donoghue M., Morrow D.A., Cannon C.P., Guo W., Murphy S.A., Gibson C.M., Sabatine M.S. (2008). Association between baseline neutrophil count, clopidogrel therapy, and clinical and angiographic outcomes in patients with ST-elevation myocardial infarction receiving fibrinolytic therapy. Eur. Heart J..

[13] Kyne L., Hausdorff J.M., Knight E., Dukas L., Azhar G., Wei J.Y. (2000). Neutrophilia and congestive heart failure after acute myocardial infarction. Am. Heart J..

[14] Menon V., Lessard D., Yarzebski J., Furman M.I., Gore J.M., Goldberg R.J. (2003). Leukocytosis and adverse hospital outcomes after acute myocardial infarction. Am. J. Cardiol..

[15] Carobbio A., Ferrari A., Masciulli A., Ghirardi A., Barosi G., Barbui T. (2019). Leukocytosis and thrombosis in essential thrombocythemia and polycythemia vera: A systematic review and meta-analysis. Blood Adv..

[16] Lawton J.S., Tamis-Holland J.E., Bangalore S., Bates E.R., Beckie T.M., Bischoff J.M., Bittl J.A., Cohen M.G., DiMaio J.M., Don C.W. (2022). 2021 ACC/AHA/SCAI Guideline for Coronary Artery Revascularization: Executive Summary: A Report of the American College of Cardiology/American Heart Association Joint Committee on Clinical Practice Guidelines. Circulation.

[17] Zwicker J.I., Paranagama D., Lessen D.S., Colucci P.M., Grunwald M.R. (2021). Hemorrhage in patients with polycythemia vera receiving aspirin with an anticoagulant: A prospective, observational study. Haematologica.

[18] Gangat N., Caramazza D., Vaidya R., George G., Begna K., Schwager S., Van Dyke D., Hanson C., Wu W., Pardanani A. (2011). DIPSS plus: A refined Dynamic International Prognostic Scoring System for primary myelofibrosis that incorporates prognostic information from karyotype, platelet count, and transfusion status. J. Clin. Oncol..

[19] Tefferi A., Rumi E., Finazzi G., Gisslinger H., Vannucchi A.M., Rodeghiero F., Randi M.L., Vaidya R., Cazzola M., Rambaldi A. (2013). Survival and prognosis among 1545 patients with contemporary polycythemia vera: An international study. Leukemia.

